# Supplementary material for: Orchid NAC Transcription Factors: A Focused Analysis of CUPULIFORMIS Genes
Source: Genes (Basel). 2022 Dec 5;13(12):2293. doi: 10.3390/genes13122293 (PMC9777940; doi:10.3390/genes13122293)
Supplement: Supplementary file 1 [file genes-13-02293-s001.zip › genes-2067628-supplementary - There is no Figure S1 here, please add/Supplementary Table S1.docx]

| **Species** | **All** | **NAM** | **NAC** | **OsNAC7** | **OsNAC8** | | **TIP** | **ANAC011** | **NAC2** | **ATAF** | **NAP** | **SENU5** | **ONAC22** | **TERN** | **ONAC003** | **ANAC063** | **ANAC001** | **AtNAC3** |
| --- | --- | --- | --- | --- | --- | --- | --- | --- | --- | --- | --- | --- | --- | --- | --- | --- | --- | --- |
| *Arabidopsis*  *thaliana* | 111 | 13 | 3 | 13 | | 3 | 4 | 8 | 10 | 4 | 5 | 2 | 6 | 2 | 7 | 19 | 9 | 3 |
| *Apostasia*  *shenzenica* | 60 | 9 | 4 | 7 | | 2 | 1 | 3 | 2 | 5 | 10 | 4 | 6 | 1 | 4 | 2 | - | - |
| *Cypripedium*  *formosanum* | 47 | 8 | 7 | 1 | | 1 | 1 | 1 | 2 | 5 | 5 | 3 | 5 | - | 6 | 2 | - | - |
| *Dendrobium*  *catenatum* | 74 | 14 | 4 | 10 | | 2 | 3 | 3 | 1 | 3 | 13 | 4 | 9 | 1 | 6 | 1 | - | - |
| *Orchis*  *italica* | 38 | 9 | 3 | 4 | | - | - | 7 | 3 | 4 | 3 | 1 | 1 | - | 2 | 1 | - | - |
| *Phalaenopsis*  *aphrodite* | 75 | 13 | 4 | 7 | | 2 | 1 | 3 | 3 | 4 | 14 | 2 | 8 | 1 | 7 | 2 | - | - |
| *Phalaenopsis*  *equestris* | 71 | 12 | 4 | 8 | | 2 | 1 | 3 | 1 | 3 | 14 | 2 | 8 | 1 | 7 | 3 | - | - |
| *Vanilla*  *planifolia* | 65 | 11 | 6 | 3 | | 1 | 3 | 1 | 3 | 3 | 6 | 2 | 13 | 1 | 10 | 2 | - | - |
| *Antirrhinum*  *majus* | 77 | 10 | 4 | 13 | | 3 | 2 | 5 | 8 | 6 | 5 | 2 | 4 | 5 | 6 | 4 | - | - |
| *Asparagus*  *officinalis* | 83 | 11 | 7 | 11 | | 1 | 2 | 7 | 5 | 5 | 8 | 5 | 8 | 1 | 5 | 17 | - | - |
| *Amborella*  *tricopoda* | 42 | 7 | 2 | 5 | | - | 2 | 4 | 2 | 3 | 4 | 1 | 5 | 2 | 3 | 2 | - | - |

**Supplementary** **Table S1.** List of the amount of genes isolated in all species analysed in this work.


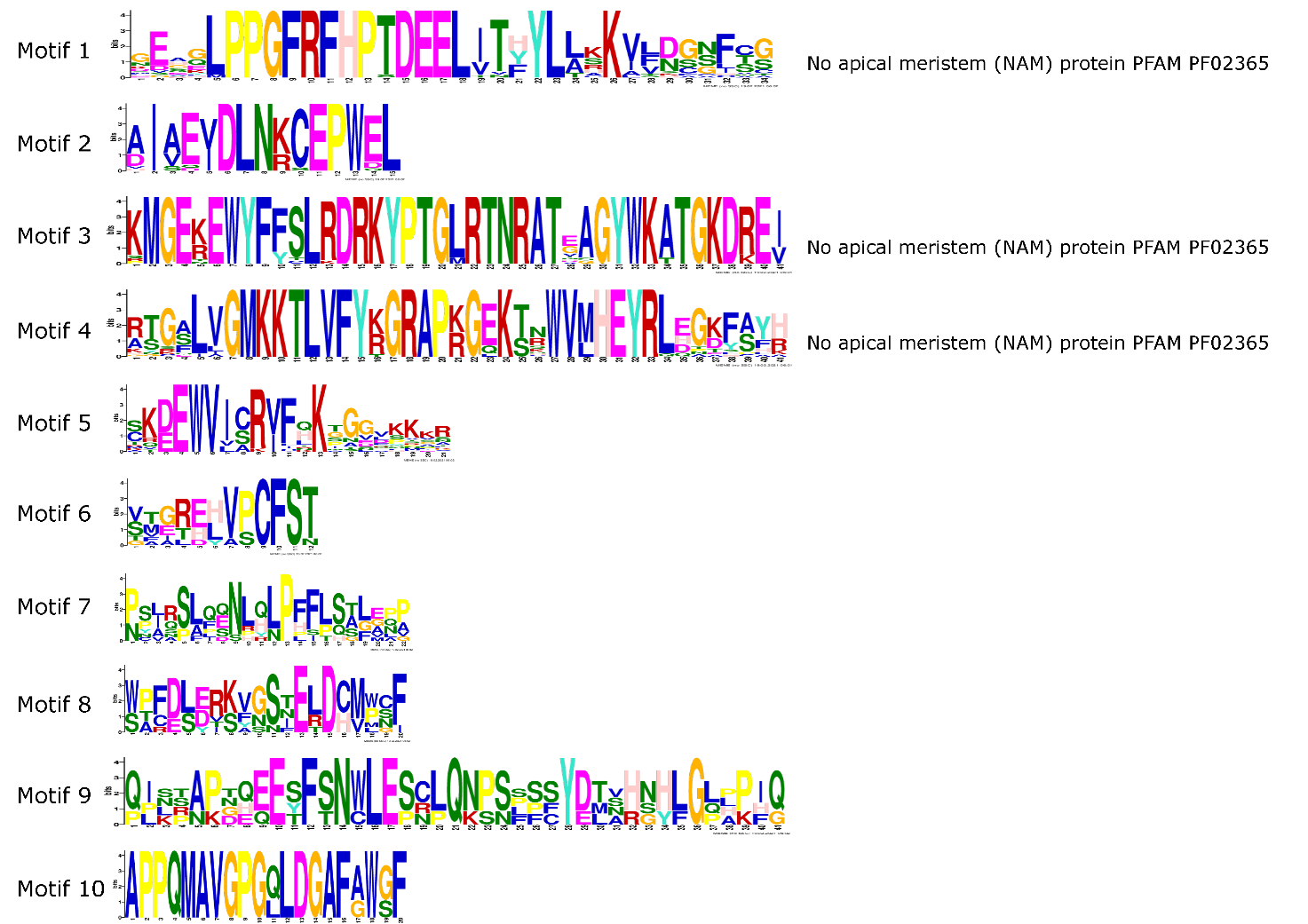


**Supplementary** **Figure S1.** Conserved motif of CUP proteins identified by MEME analysis.
